# New insights into the antibacterial mode of action of quercetin against uropathogen *Serratia marcescens* in-vivo and in-vitro

**DOI:** 10.1038/s41598-022-26621-0

**Published:** 2022-12-19

**Authors:** Suganya Kannan, Jeyakumar Balakrishnan, Ambujam Govindasamy, R. Arunagiri

**Affiliations:** 1 Central Research Laboratory, Vinayaka Mission’s Medical College and Hospital, Vinayaka Mission’s Research Foundation (Deemed to be University), Karaikal, India; 2 Department of General Surgery, Vinayaka Mission’s Medical College and Hospital, Vinayaka Mission’s Research Foundation (Deemed to be University), Karaikal, India

**Keywords:** Antimicrobials, Applied microbiology, Pathogens

## Abstract

In the course of a quest for therapeutic agents inhibiting uropathogens, the rise and universal blowout of antibiotic-resistant organisms is a wide problem. To overcome this matter, exploration of alternative antimicrobials is necessary. The antimicrobial potential of quercetin has been widely described against some pathogenic microorganisms, but to the best of our knowledge, no report exists against the pathogenicity of uropathogenic *Serratia marcescens*. Hence, the present study focused on the antibacterial mechanism of action of quercetin, a flavonoid against the uropathogen *Serratia marcescens*. Quercetin was evaluated for its anti-QS activity, and the attained outcomes showed that quercetin inhibited QS-mediated virulence factors such as biofilm formation, exopolysaccharides, swarming motility and prodigiosin in *Serratia marcescens*. The proposed mechanism of action of quercetin greatly influences cell metabolism and extracellular polysaccharide synthesis and damages the cell membrane, as revealed through global metabolome profiling. In vivo experiments revealed that treatment with quercetin prolonged the life expectancy of infected *Caenorhabditis elegans* and reduced the colonization of *Serratia marcescens.* Hence, the current study reveals the use of quercetin as a probable substitute for traditional antibiotics in the treatment of uropathogen infections driven by biofilms.

## Introduction

*Serratia marcescens* (SM) is a recently emerged nosocomial pathogen that causes a wide range of ailments, such as surgical wound infections, UTIs and septicemia. *S. marcescens*, has long been considered as a harmless commensal but it has been now recognized as a frequent cause of nosocomial extra intestinal infection. Unfavorably, an apparent upsurge in antibiotic resistance by SM is now becoming life-threatening^1–5^. The potential of nosocomial pathogens to attach abiotic and biotic superficial is an extensive intrinsic factor accounting for catheter-related sepsis globally. Biofilms are composed of a self-synthesized hydrated extracellular polymeric matrix made up of bacterial mass and essential biomolecules^6^.

The biofilm mode of life in SM enables a protected manner of growth that allows the survival of bacteria in a condition of danger such as antibiotics or a host immune system^7^. It has been known well that in SM the quorum sensing (QS) regulates the expression of genes responsible for the synthesis of virulence factors such as hemolysin, proteases, chitinase, chloroperoxidase, alkaline phosphatase, the ability to swim, swarm and biofilm maturation. Moreover, SM synthesizes a red pigment called prodigiosin (2-methyl-3-pentyl-6-methoxyprodigiosin), which has a wide range of virulence competencies^8,9^.

As a result of the parched antibiotic finding pipeline, the consideration of flavonoids seems to give some promising hope. In certain instances, the antibacterial action of flavonoids has been six times greater than those of normal drugs on the market^10,11^. Quercetin is a flavonol group of polyphenolic bioflavonoids found in fruits and vegetables. Quercetin has been well known for its antimicrobial, antioxidant, anti-inflammatory, antitumor and other biological activities. However, studies on its anti-biofilm properties and its influence on the progress of adaptive resistance in SM remain scanty^12,13^.

The exact mode of antimicrobial action of quercetin against SM was not completely pictured. Metabolomics affords the chance to achieve a scheme eclectic portrait of biochemical and cellular complexes under well-defined conditions^14^. In recent years, the metabolomics-based approach has been widely applied in the field of drug research to elucidate the mechanism of drug action. Moreover, thorough knowledge of cellular metabolic trepidations during the treatment of antimicrobial agents can facilitate the discovery of innovative drug targets. Here, we performed an untargeted metabolomics investigation to explicate the mechanism of quercetin killing against SM. This is the first study to disclose the metabolic perturbations prompted by quercetin treatment. Our data deliver a novel understanding into the machinery of killing against SM. The traditional pipeline for antimicrobial drug innovation typically originates with in vitro screening of test compounds subsequently imperiling potential hits to in vivo animal testing^15^. Conversely, these prospective compounds frequently turn out unveiling poor pharmacokinetic actions and/or are exceedingly toxic when verified in vivo. To overcome these restrictions, an alternate way is to conduct the preliminary testing of compounds in the C*. elegans* whole animal system. Revealing worms to both pathogen and test compounds and consequently observing worm survival through the assay can be certainly accomplished as an alternative for antimicrobial screening of test compounds in vivo.

## Materials and methods

### Medium and culture conditions

The strain *S. marcescens* ATCC 14756 was used in this study. The strain was cultivated and maintained in tryptone soy (TS) broth with a pH of 7.0 at 37 °C overnight. For the virulence assays, the strain was subcultured in TS broth up to an OD of 0.5 at 600 nm. For the development of biofilms, brain heart infusion (BHI) broth was adapted^16,17^.

### Preparation of compound

Quercetin was purchased from Sigma-Aldrich and dissolved in DMSO at a concentration of 5 mg mL^−1^ as a stock, and the preparations were stored at 4 °C.

### Determination of MIC of quercetin

MIC was calculated using the Clinical and Laboratory Standards Institute procedure (CLSI) with the broth microFdilution assay. In the wells of the microdilution plate, 1% of the *S. marcescens* ATCC 14756 inoculum (OD = 0.5) was added to the MH broth and mixed well. Subsequently, quercetin was added to the inoculum at concentrations of 5 to 500 µg mL^−1^. After incubation of plates at 37 °C for 24 h, the OD_600_ was recorded with a SpectraMax plus 96 microplate reader. The lowermost concentration of quercetin where there is no turbidity was documented as the MIC^18^.

### Inhibition of extra polymeric substances

EPS was quantified with a total polysaccharide quantification assay^19^. SM was grown in the presence and absence of quercetin in wells of 24-well microtiter plates supplemented with 1 mL of BHI medium to promote adherence and EPS production. After 16 h to 48 h of incubation at 37 °C, the excess medium was removed, and the adherent film was rinsed with PBS to eliminate the unattached planktonic biomass. Subsequently, 0.5 mL of 1% phenol and 5 volumes of concentrated H_2_SO_4_ were added to the wells and incubated in the dark for 1 h. After incubation OD_490_ was measured and the % EPS inhibition was calculated.

### Effect of quercetin on the biofilm formation of SM

SM cells were harvested during the stationary phase and suspended in PBS, and the OD_600_ was adjusted to 0.5. The dilutions were made in BHI broth at a ratio of 1:100. The suspension was loaded in the wells of a microliter plate with and without quercetin (½ MIC, ¼ MIC) and incubated at 37 °C for 24 to 48 h. After incubation, the excess medium was removed and the planktonic cells were removed by washing with PBS. The developed film was stained with 0.2% crystal violet for 5 min, the additional stain was discarded, and the bound film was eroded with PBS. For the quantification of biofilm, the adhered dye was solubilized with 75% ethanol. The unbound dye was quantified by determining the OD_650_ and comparing it with the control. The % inhibition was calculated according to the method of de Oliveira et al.^20^.

### Quantification of metabolically active cells by MTT reduction

The effect of quercetin on SM cell viability and metabolic activity was calculated using 3-(4,5-dimethylthiazol-2-yl)-2,5-diphenyltetrazolium bromide (MTT) reduction. In both control and treated wells, 50 μL of MTT (1 mg mL^−1^) solution was added and allowed for the development of formazan crystals in the dark for approximately 5 h at 37 °C. After incubation, the crystals were solubilized with 100 µL of isopropanol for 1 h and the absorbance was measured at OD_570_ using a SpectraMax plus 96 microplate reader. The % inhibition was calculated by using the following formula.

$$ \% {\text{ inhibition }}\left( {\text{c}} \right) \, = \, [{1} - ({\text{A}}_{{\text{C}}} /{\text{A}}_{0} )] \, \times { 1}00. $$

Here A_c_ is the absorption of each well^21^.

### Inhibition of virulence factor regulated by QS

#### Swarming motility inhibition

To evaluate the pattern of swarming motility, 5 µL of SM (OD adjusted to 0.4 at 600 nm) cells was point inoculated at the center of the swarming agar medium (1% peptone, 0.5% NaCl, 0.5% agar and 0.5% of filter-sterilized d-glucose) prepared with and without quercetin at sub MIC concentrations. The plates were incubated at 37 °C in an upright position for 18 h and observed for reduction in swarming migration zones^22^.

#### Quantification of prodigiosin

The effect of quercetin on prodigiosin pigment synthesis was evaluated according to the technique of Gowrishankar et al. LB medium was inoculated with 1 × 10^10^ CFU mL^−1^ of SM cells with and without quercetin, and the suspension was incubated at 35 °C for 24 h. After incubation, cells were harvested by centrifugation at 12,000 rpm for 10 min. To extract prodigiosin from the pellet, 1 mL of acidified ethanol (4% of 1 M HCl added with ethanol) was added and mixed well.The resultant supernatant containing prodigiosin was measured spectroscopically at 534 nm and the % inhibition was calculated from the absorbance values^23,24^.

#### Confocal laser scanning microscopy analysis of SM biofilm matrix architecture

For confocal imaging of SM biofilms, the cells were grown with and without the presence of quercetin above the glass coverslip substratum kept in 24-well Corning Costar TC plates flooded with BHI broth. The biofilms were allowed to develop on coverslips for 24 h to 48 h at 37 °C. Detachment of planktonic cells was performed with the help of a subsequent gentle rinse with PBS. After incubation, biofilms were stained with 50 mM acridine orange and washed to remove any remaining stain. Consecutively, the dried coverslips were visualized under CLSM (LSM 710, Carl Zeiss, Germany), with excitation filter of 515–560 nm and the Z stack analysis was performed using Zen 2009 156 software (Carl Zeiss, Germany)^25^.

#### Scanning electron microscopy analysis of biofilm formation

The cell morphology of biofilms developed on glass slides with and without quercetin treatment was assessed by SEM. SM biofilms were endorsed to develop on pre-sterilized glass surfaces (1 cm × 1 cm) in 6-well cell culture plates (Tarsons, India). The effects of quercetin incubation and the dispersion of pre-established biofilms were monitored by SEM as previously explained. Biofilms without quercetin treatment served as controls. Dehydrated slides were sputtered with a gold coating for enhanced imaging (20 min, 20 mA; Q150R Quorum Technologies, Lewes, UK). The sputter-coated specimens weretested using a scanning electron microscope with accelerating voltages of 1, 5, 10 and 20 kV (JSM-6390LV, JEOL, and Tokyo, Japan). The magnifications used were × 1500 and × 5000^26^.

### Quantitative real time PCR (qPCR)

SM cells treated or untreated with quercetin were lysed with Tris–EDTA and RNA lysis buffer. To isolate total RNA, TRIzol reagent (Thermo Scientific) was used, and the DNA was removed by DNase-I treatment. The concentrations of RNA were quantified with a NanoDrop spectrophotometer and stored at − 80 °C. Reverse transcription of the RNA was performed using Revertaid reverse transcriptase (Thermo Scientific) and qPCR was performed using a real-time PCR system (Quant studio 5, Applied Biosystems). The effects of quercetin at sub-MIC concentrations on the expression levels of SM virulence s responsible for motility (*rsmA flhC* and *flhD*), prodigiosin synthesis (*pigB*), pore forming toxin (*shlA*) and biofilm formation (bsmA and *bsmB*) were evaluated.

### Metabolomic and transcriptomic variation with quercetin treatment

#### Metabolite extraction from SM

A 10 mL overnight culture of SM was inoculated into 90 mL of Tryptone soy broth, and the mixture was incubated with shaking at 37 °C. Quercetin stress was given at ½ × MIC concentration, and samples were collected at 12 h after the addition of the drug. In a dry ice-ethanol bath, the cultivated culture was chilled to 5 °C to terminate metabolism quickly before it was transmitted to ice. The cells were centrifugedat 11,000 rpm, rinsed with 10 mL ice-cold PBS and resuspended in 1 mL of PBS. The OD_600_ of the cell suspension, both controlled and treated, was obtained and adapted to 1.0. One mL of cells was pelleted and suspended in 200 μL of chloroform: methanol: water (1:3:1, by volume) mixture. Samples were exposed to four freeze–thaw cycles in a dry-ice-ethanol bath with frequent vortexing. The supernatant was collected and stored under argon at − 80 °C. Metabolites were examined using an ultra-high-pressure liquid chromatography–tandem mass spectrometry (UHPLC-MS/MS) system entailing a Dionex UHPLC system combined with electrospray ionization (ESI; negative polarity) and a hybrid quadrupole high-resolution mass spectrometer (Q Exactive Orbitrap; Thermo Scientific) functioning in full scan mode to expose all the metabolites based on their exact masses and retention times^27^.

### Quercetin mediated protection of in vivo model *C. elegans* against SM

#### *C. elegans* strain and growth conditions

The strain Bristol N2 *C. elegans* was cultivated at 16 °C on nematode growth medium (NGM) inoculated with a layer of *E. coli* OP50 strain at the center of the plate, which was prepared as previously described^28^.

#### Lifespan assays

*Caenorhabditis elegans* longevity was evaluated by inoculating 50 μL of *E. coli* OP50, and SM cultured in BHI broth on an NGM agar plate and incubated overnight at 37 °C. The experiment was carried out in three groups: Group I—worms fed with *E. coli* OP50, Group II—worms fed with SM, and Group III—worms fed with quercetin exposed SM. L4 stage young adults of worms (n = 30) were seeded on the respective plates and scored for live and dead every 12 h. Live worms were relocated into fresh NGM agar plates seeded with the appropriate treatment group every 48 h. Each of the plates was monitored until all the worms were dead. After incubation, the mean life span (MLS) was calculated according to the method of kamaladevi et al.^29^.

#### Microscopic observation of physiological defects

The treated and untreated groups of worms were rinsed thrice with M9 buffer and kept on a 2% agarose pad with 1 mM sodium azide to observe under an inverted fluorescence microscope (Nikon, Japan).

#### Bacterial colonization assay

The impact of quercetin on the colonization of C*. elegans* exposed to SM with and without the drug combination was evaluated after 24 h of colonization. The untreated control and treated nematodes were rinsed with M9 buffer to remove the surface adherent bacterial cells. Furthermore, the motile worms were stained with 10 µM acridine orange for 10 min, anesthetized with 1 mM sodium azide and visualized under a fluorescence microscope. For the quantitative analysis of colonization in the gut, the worms were mechanically disrupted in a 1.5 mL centrifugal tube containing 50 μL PBS with 1% Triton X-100 using a micro pestle. Worm lysates were then diluted in PBS and plated on LB agar at 37 °C for 24 h. The colonies were enumerated after incubation and the CFU per nematode was calculated Beale et al.^30^.

### Data analysis and statistics

The metabolomics data generated were subsequently imported into MPP software (version B.12.61) for binning, aligning, and creating a consensus for each feature. Statistical differences between metabolomic data were investigated by one-way ANOVA followed by Tukey’s HSD post hoc test and FDR correction using MetaboAnalyst.

## Results

### Antimicrobial activity of quercetin against SMplanktonic cells

Primarily, the effect of quercetin on the growth of SM planktonic cells was evaluated at concentrations from 5 to 500 μg mL^−1^, in which growth inhibition was initiated at the minimum concentration. As a result, the MIC and MBC values of quercetin were recorded as 175 μg mL^−1^ and 325 μg mL^−1^, respectively.

### Effect of quercetin on biofilm formation

In addition to growth inhibition, quercetin has also been assayed for antibiofilm activity against both early and mature biofilms in a dose-dependent manner. The planktonic cells were grown in the wells of micro titer plates for 12 to 48 h with and without quercetin (¼ MIC and ½ MIC).

**Figure 1:**
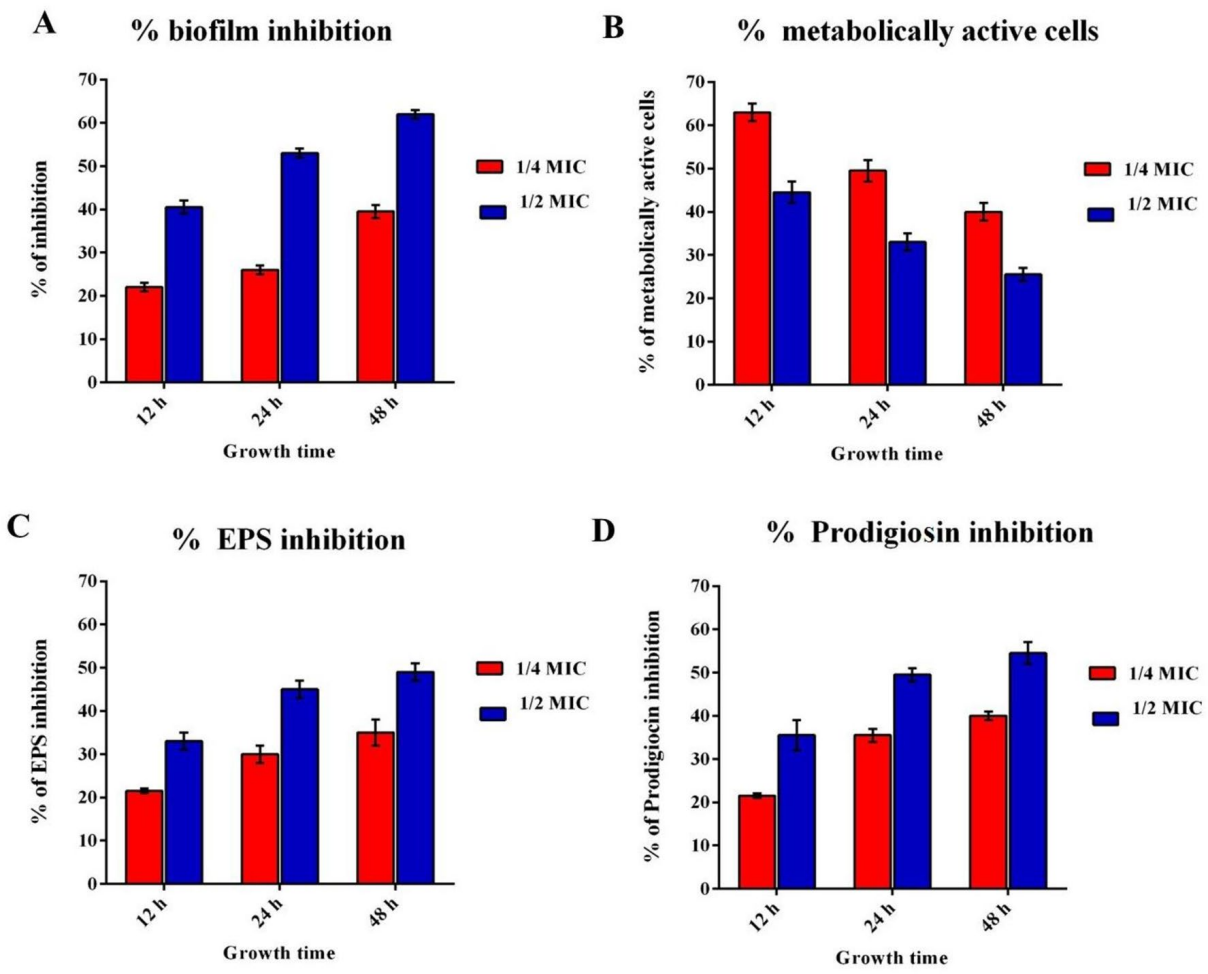
Percentage of inhibition of (**A**) Biofilm production (**B**) metabolic activity (**C**) EPS production (**D**) Prodigiosin synthesis exerted by quercetin on *Serratia marcescens*. All the assays were done in triplicates and the values were expressed as mean ± SD.

The overall biofilm biomass was measured by crystal violet assay with the sub-MIC concentration of quercetin. SM biofilm inhibition was effectively recorded with the treatment of quercetin. SM developed a dense layer of biofilm in control wells, whereas in the presence of quercetin, the biofilm inhibition was recorded as 44% at ½ MIC and 21% at ¼ MIC in young 12 h biofilms. Similarly, the inhibition rate was significant in both mature 24 h biofilms and old 48 h biofilms. The results showed that quercetin was effective against young, mature and old biofilms (Fig. 1A).

### Quantification of metabolically active cells by MTT reduction

The metabolic activity and viability of SM cells were evaluated by the reduction of 3-(4,5-dimethylthiazol-2-yl)-2,5-diphenyltetrazolium bromide (MTT). The MTT assay confirmed the results of the biofilm inhibition assay, as quercetin considerably exterminated the sessile cells of SM in a dose-dependent manner in all biofilm stages. Quercetin at ¼ MIC and ½ MIC efficiently reduced the viability of preformed biofilm cells. The results were further confirmed with the findings of crystal violet staining for biofilms showing a significant reduction in stained biomass with the presence of quercetin (Fig. 1B).

### Inhibition of EPS

EPS is a critical mass of biofilms and is significant for cell adherence, biofilm construction and protection of biofilm residing pathogenic cells. Henceforth, the effect of quercetin on the EPS production of SM was evaluated. The results showed that quercetin at ½ MIC and ¼ MIC considerably repressed EPS assembly and synthesis to 35% and 22% in the 12 h grown film. The same effect was observed in the highly dense 24 h and 48 h EPS matrix of the bacterial biofilm (Fig. 1C).

### Inhibition of prodigiosin pigmentation

**Figure 2:**
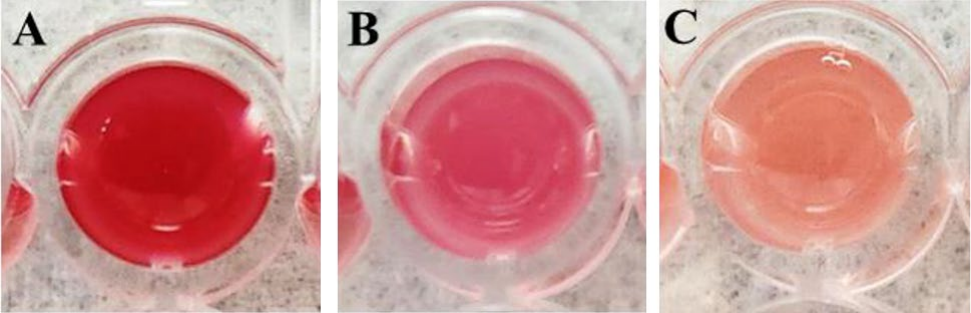
Red-pigmented cultures with various concentrations of quercetin. Dose response showing inhibitory effects of quercetin on prodigiosin production in *S. marcescens* (**A**) Control, (**B**) treated with ¼ MIC, (**C**) with ½ MIC. Intracellular prodigiosin was extracted and quantified. The results were reproduced in three experiments, and the error bars indicate standard deviations.

Substantial suppression of the formation of prodigiosin pigment was observed with quercetin concentrations of ¼ MIC and ½ MICat 21% and 34%, respectively with 12 h of treatment (Figs. 1D, 2). Quercetin was found to be an effective inhibitor for the synthesis of prodigiosin to 55% at a final concentration of ½ MIC. The findings showed that quercetin reduced the synthesis of prodigiosin in both the dose and time dependent manner.

### Inhibition of swarming motility

**Figure 3:**
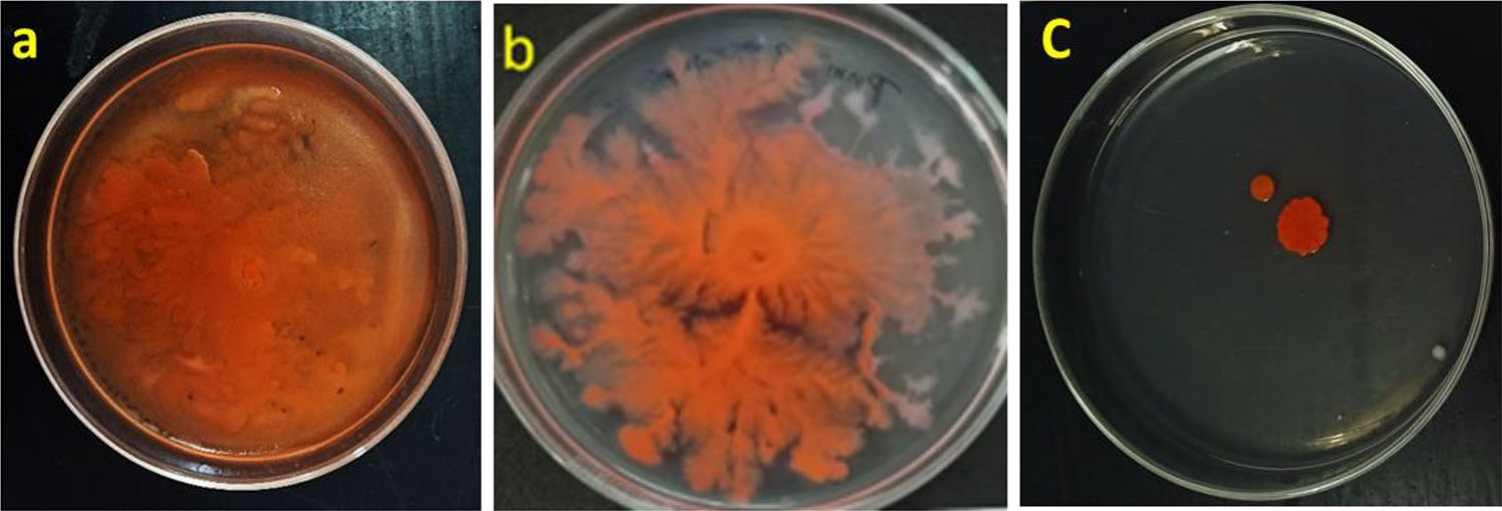
Effect of quercetin on the swarming motility of *S. marcescens* (**a**) untreated control, (**b**) ¼ MIC treated, (**c**) ½ MIC treated.

Swarming motility is a typical SM virulence mechanism that has a powerful role in catheter associated urinary tract infection. The ability of SM to spread across the swarm agar plate has thus been examined in the presence of quercetin. Quercetin remarkably reduced SM swarming motility in comparison to its various controls. The motility pattern and substrate coverage were greatly inhibited by the presence of quercetin treatment (Fig. 3). The average (n = 3) of the swarming diameters after 20 h of incubation in the absence and presence of quercetin were recorded as 30 mm and 12 mm, respectively.

### Microscopic observation of the antibiofilm potential of quercetin

**Figure 4:**
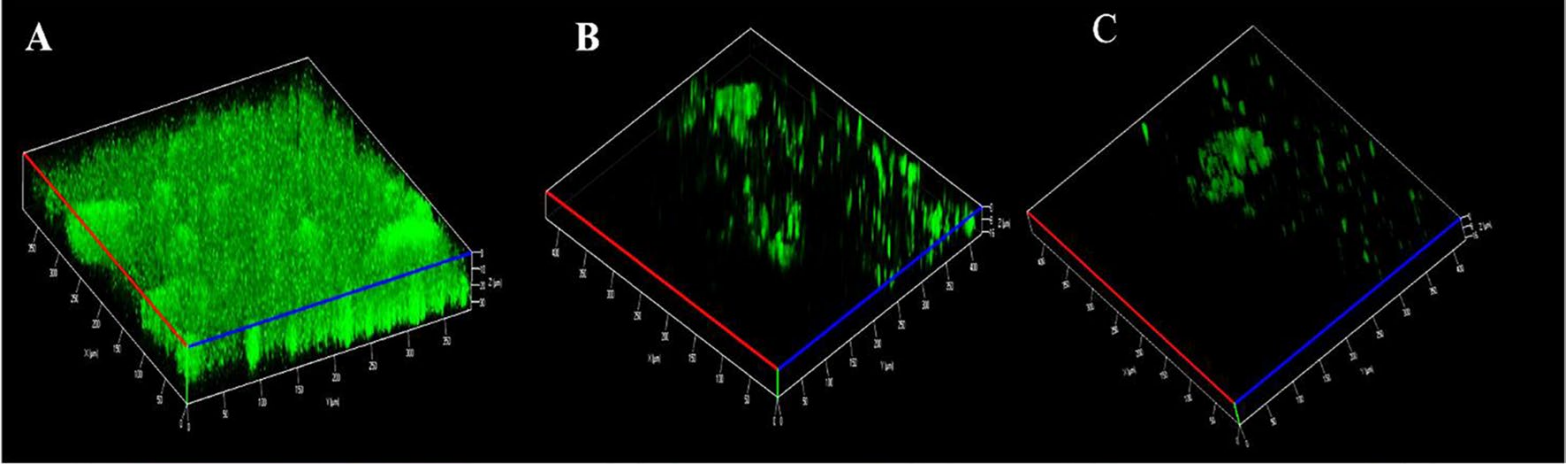
CLSM microscopic analyses of *S. marcescens* biofilm formation. (**A**) Control untreated biofilm. (**B,C**) showed disintegration of *S. marcescens* biofilm formation compared to their untreated controls.

**Figure 5:**
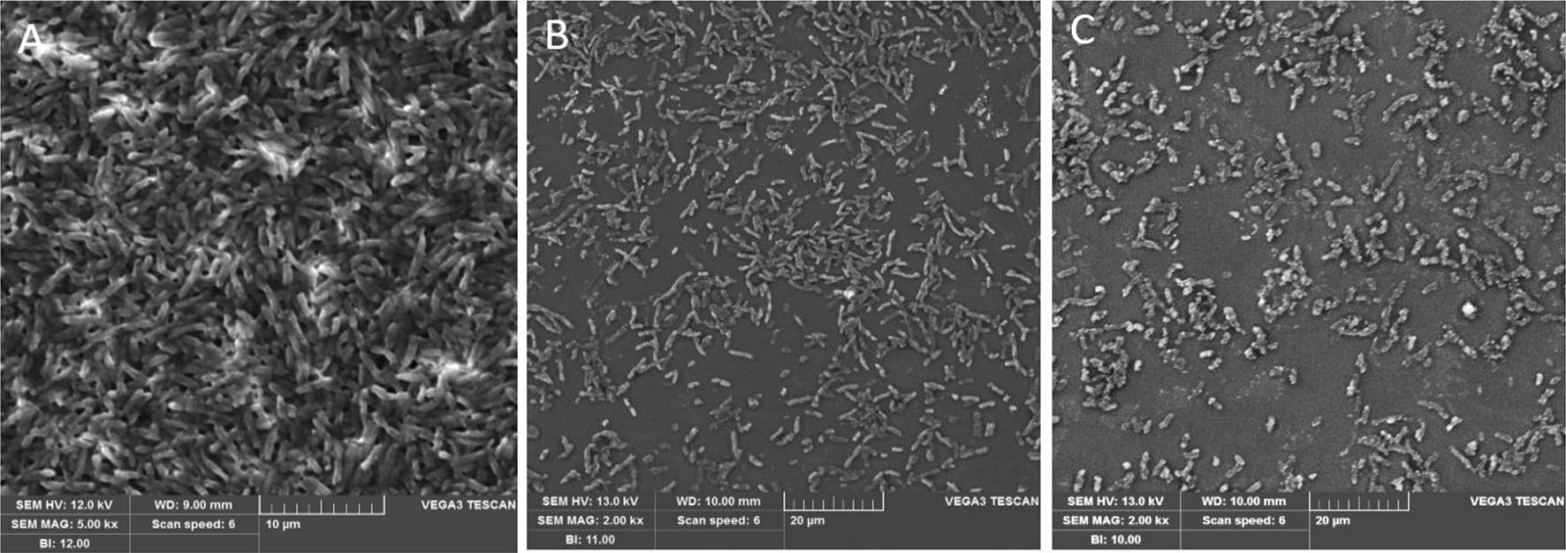
Scanning electron microscopy images of *S. marcescens* biofilm. (**A**) Control shows biofilm formation after 24 h of incubation. (**B,C**) Quercetin inhibited the biofilm formation at a concentration of ¼ MIC and ½ MIC.

The antibiofilm efficacy of quercetin on biofilm development was confirmed through microscopic techniques such as SEM and CLSM (Figs. 4, 5). The results of the CLSM and SEM images revealed a high surface coverage of biofilm formation in control slides, while in treatment slides, a visible decline in biofilm progress was observed. In the quercetin treated sample, irregular shapes due to severe cellular destruction, reduced EPS production, and scattered microcolonies were observed. In contrast, the untreated cells exhibited an impenetrable and elevated surface-covered biofilm with a dense layer of EPS in both SEM and CLSM analysis.

### Quantitative real time PCR (q-PCR)

**Figure 6:**
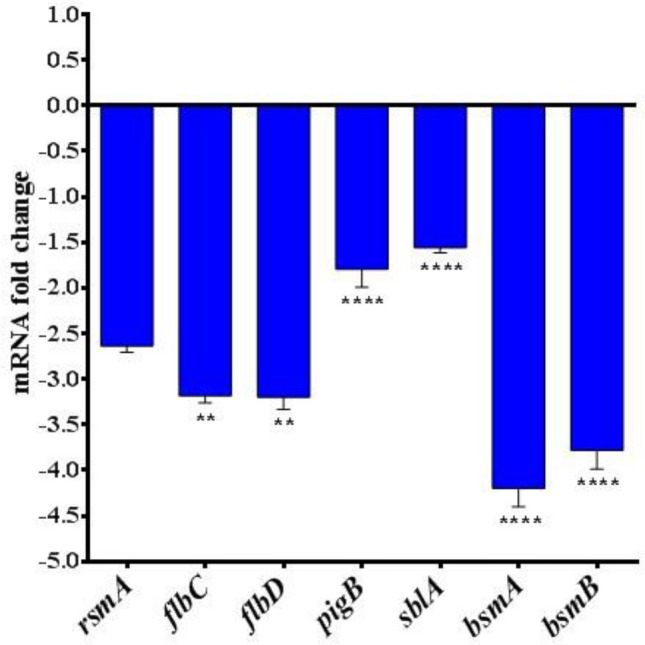
Downregulation of *S. marcescens* virulence genes.

qPCR was performed to assess the effects ofquercetin on the transcriptional levels of the QS-mediated genes *rsmA, flbC, flbD, pigB, sblA, bsmA,* and *bsmB*, which are responsible for swarming and swimming motility, pigment production, adherence, and biofilm formation, respectively. The gene expression patterns of both quercetin-treated and control SM cells were evaluated by usingthe 2^−ΔΔCt^ method. The expression levels of all the genes (*rsmA, flbC, flbD*, *pigB, sblA, bsmA, and bsmB*) were considerably decreased compared to untreated control cells (Fig. 6). The data are presented as the mean ± standard error from three independent experiments, and *P* < 0.05 was considered significant using Student’s t-test.The expression levels of swarming motility genes were abridged by 2.4-fold for *rsmA* in the presence of quercetin at sub-MIC concentrations. In addition, quercetin at sub-MIC concentrations decreased the expression of the prodigiosin-encoding gene *pigB* by 1.5-fold.

### Untargeted metabolomics to ascertain the modes of action of quercetin against *S. marcescens*

Metabolomic investigation revealed distinct metabolic reprogramming during disease progression, which was significantly altered in the treated population. From the score plot resulting from PLS-DA analysis of metabolome data collected by the LC/MS/MS system, it is evident that principal component 1 (PC-1) accounted for 31.3% of the variance, with principal component 2(PC-2) accounting for 34%.

**Figure 7:**
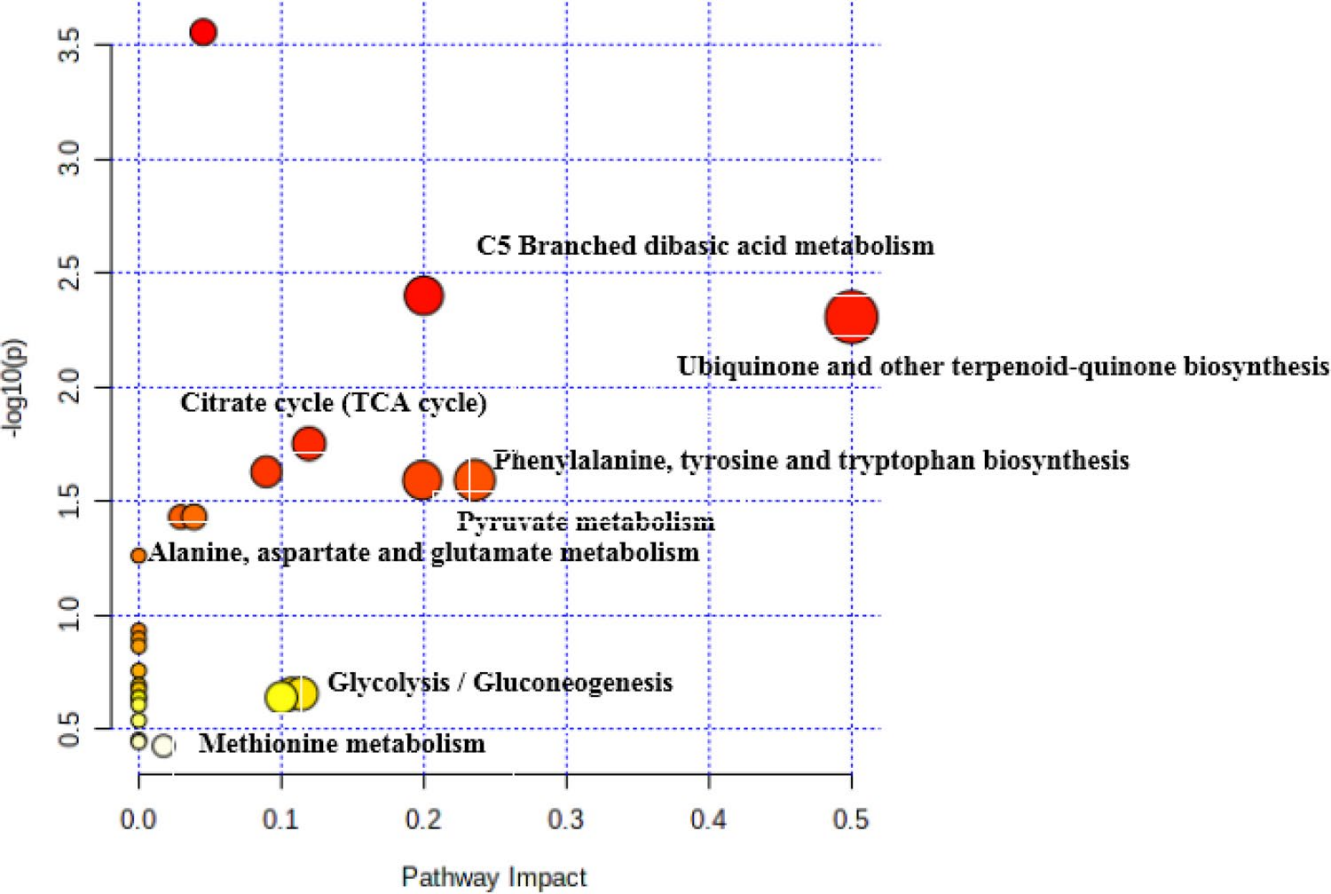
Metabolomic pathway analysis of Quercetin treated *Serratia marcescens* metabolome by MetaboAnalyst 3.0 Software according to KEGG pathway database. The darker the color and larger the size represent higher P-value from enrichment analysis and greater impact from the pathway topology analysis, respectively.

The number of metabolic features detected was between 790 and 2752 base peaks and 7929 and 1,516 metabolite for which a putative annotation could be given. Metabolic reprogramming is supposed to be the keydriving factor for biofilm formation even though such organisms are structured by the involvement of multiple layer molecules. We first demonstrated obvious modifications in small molecule metabolism and morphological features between quercetin-treated and untreated cells; however, the profound metabolic mechanism remains largely unexplored. Here, we further combined open-source databases with local databases to identify the pivotal metabolites and associated metabolic pathways that were significantly altered during quercetin as an antibiotic stress. Unexpectedly, we successfully identified 29 differential metabolites, including amino acids, carbohydrates, organic acids, glycerol-derived compounds, polyamines and uridine, between quercetin-treated and untreated cell populations. Remarkably, glycerol-derived metabolites (2-phosphoglyceric acid, glycerol 3-phosphate and d-glyceraldehyde 3-phosphate) were significantly upregulated in the untreated population, while they were decreased considerably in the treated set of cells (Fig. 7). Next, to home those differential metabolites to their metabolic pathways, we subjected them to Metaboanalyst online to annotate associated metabolic pathways. We conclude thatmetabolism was mostly reprogrammed and involved glycolipid metabolism, amino acid methionine metabolism and carbohydrate metabolism, which were observed to markedly drive biofilm formation, as most of the differential metabolites were down regulated in the treatment groups.

### Lifespan extension of *C. elegans* with quercetinmediated protection

**Figure 8:**
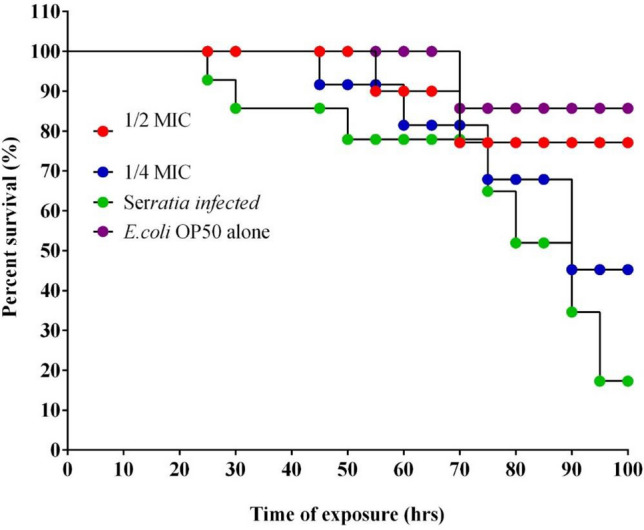
Survival curves of *C. elegans* of quercetin treated and untreated groups. The survival rates were subjected to Kaplan–Meier survival analysis to prepare survival curves and the data were compared with untreated worms. P values ≤ 0.05 were considered as significant.

Quercetin showed a better protective effect against SM infection. The average lifespan of wild-type worms in the untreated control (*E. coli* OP50 fed worms) was recorded as 15.5 days. However, the SM*-*infected group exhibited a reduced lifespan of 7.5 days. Quercetin treated SM*-*infected worms were found to have increased mean lifespan of 13.2 days when compared to SM-infected worms. Quercetin showed the highest protection by showcasing the null mortality up to 96 h in the *S. marcescens* infected group of worms. The percentage survival rate was highly restored in the presence of quercetin (Fig. 8).

### Quercetin treatment protects nematodes from SMmediated physiological impairments

**Figure 9:**
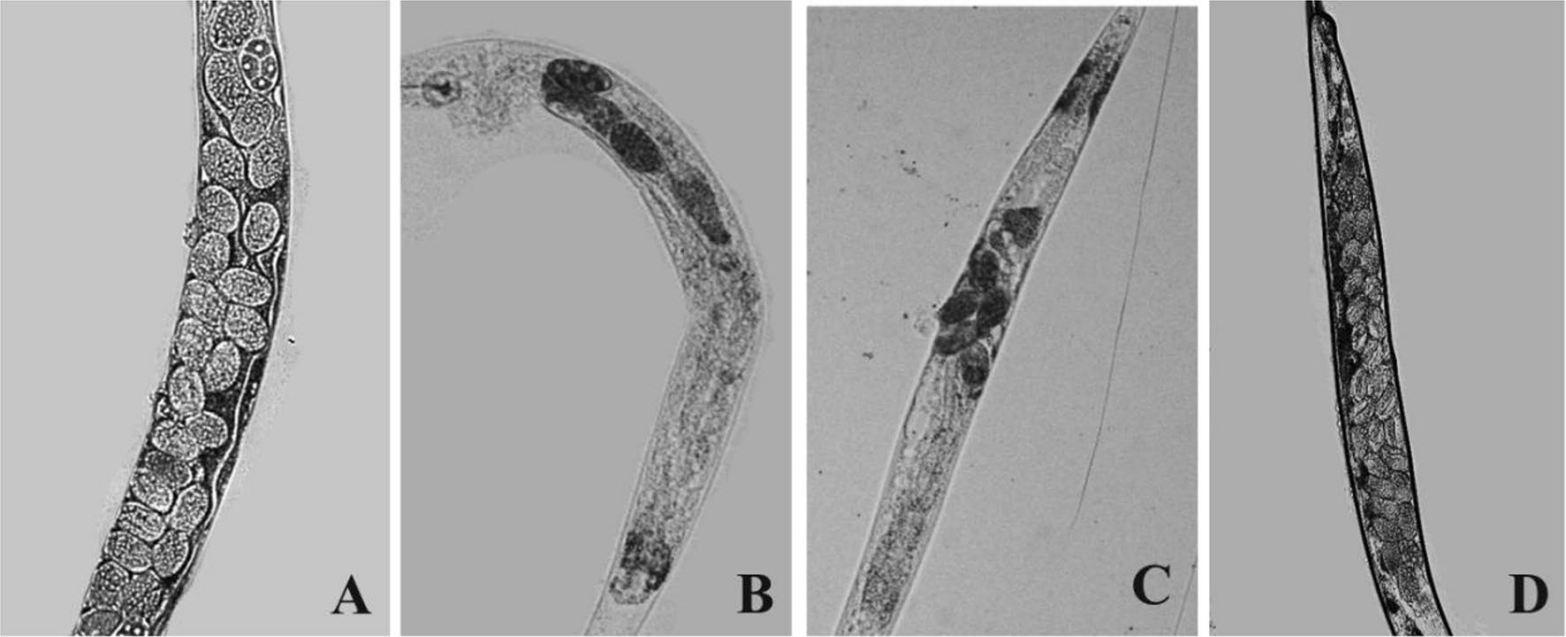
Microscopic images of the *C.* *elegans* exposed to *Serratia marcescens* for 24 h at 20 °C were presented. (**A**) Wild-type *C. elegans* fed with OP50 showed normal eggs with normal physiology. (**B**) In disparity, *Serratia marcescens* challenged nematodes exposed presented severe physiological defects such as distended pharynx and intestinal colonization (**C,D**) nematodes that was treated with quercetin showing reduced colonization and physiological damage compared to the control at ¼ MIC and ½ MIC concentration.

Infection with SM caused immature vulval development, leading to egg laying defective worms and resulting in internal hatching. Micrographs of SM*-*infected *C. elegans* revealed reproductive defects. Figure 9A shows a control *C. elegans* fed on *E. coli* OP50 with normal eggs and vulva. However, SM*-*infected worms accumulated eggs inside the uterus, which was evidenced through reduced egg laying. Additionally, it also caused distended pharynx and abnormal egg formation inside the uterus of *C. elegans* (Fig. 9B–D). Microscopic observation of control and quercetin treated *worms* displayed no such abnormality.

#### Quercetin treatment reduces in vivo intestinal colonization of SMin *C. elegans* intestine

SM produces chronic mortal infections via colonization and spreads inside worms’intestinal and urogenital tracts. To investigate this, microscopic observations and CFU tests were used to assess the quercetin treatment on SM-infection in the intestinal and urogenital regions of the nematode. Microscopic examination of the *pretreated C. elegans* clearly showed that SM colonization was decreased with the administration of quercetin.

**Figure 10:**
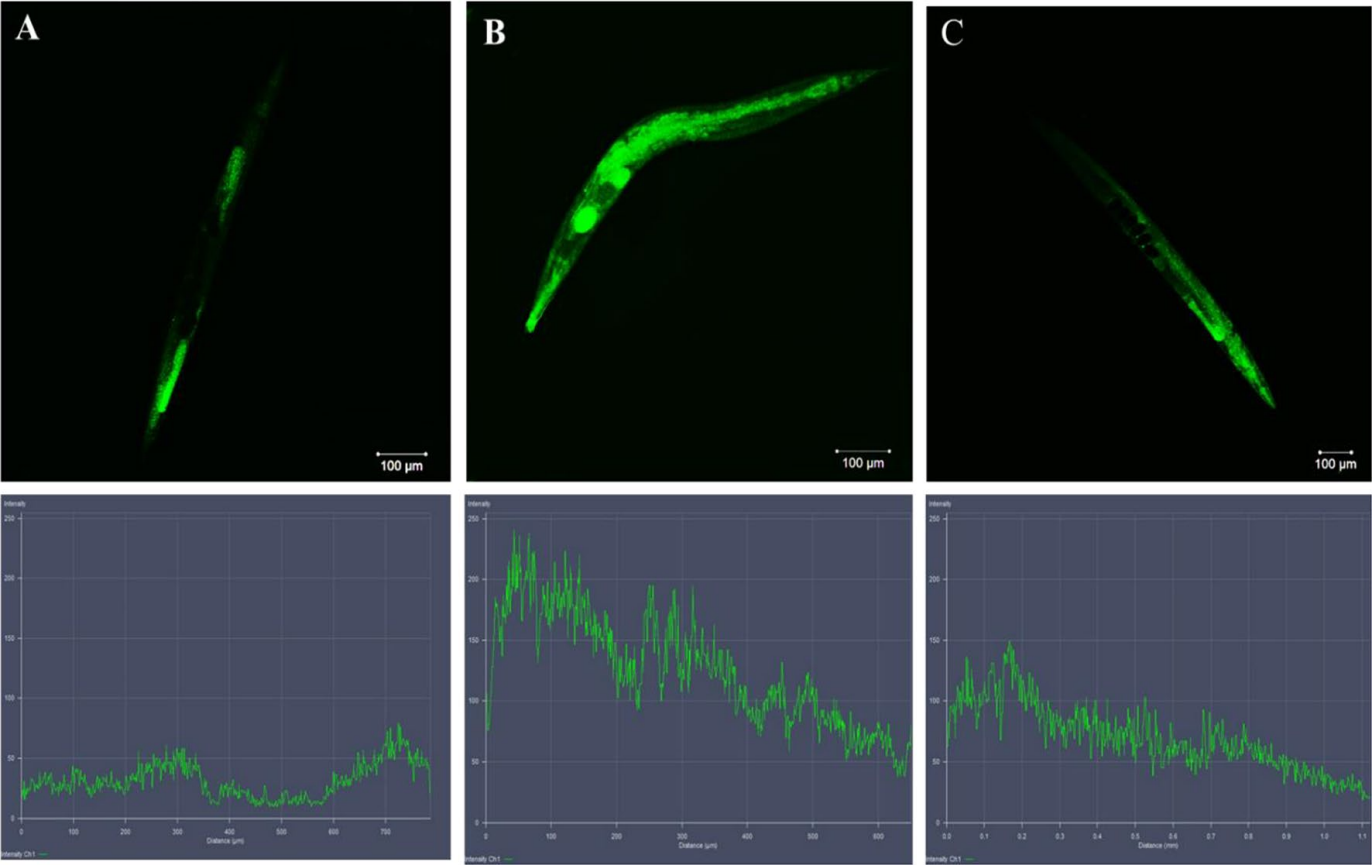
The confocal laser scanning micrographs showing the intestinal accumulation of *Serratia marcescens* in nematodes with and without quercetin by acridine orange staining method. (**A**) ½ MIC treated, (**B**) control without any treatment (**C**) ¼ MIC treated. The histogram indicates the quantitative amount of intracellular accumulation of *Serratia marcescens* in host.

The C*. elegans* exposed to SM for 24 and 48 h showed dense colonization, whereas quercetin treatment showed decreased colonization in the intestines of *C. elegans* (Fig. 10). The CFU test was conducted to further validate the microscopic findings and to assess the colonized SM. The findings showed that SM exposed nematodes for 24 h and 48 h had increased CFUs of approximately 4.27 ± 0.39 and 5.40 ± 0.40, respectively, whereas bacterial burdens were reduced to approximately 1.5 ± 0.18 and 1.63 ± 0.25 at 24 h and 48 h, respectively*.*

## Discussion

The present study demonstrated the antimicrobial and anti-infective potential of quercetin, a flavonoid derived from fruits and vegetables, against SM, which is a well-recognized opportunistic pathogen that rouses healthcare associated infections. The development of biofilm boosts the infectious disease causing potential of SM in healthcare locales and leads to the rise of antibiotic resistance. Therefore, anti-biofilm based tactics to combat infectious ailments have owned added attention among the scientific community. Quercetin is a non-toxic natural molecule, and although well known for various biological activities, the antibiofilm efficacy ofquercetin against SM remains unexplored. Previously, quercetin at MIC, i.e., a concentration of 512 mg L^−1^ stopped bacterial growth of *E. faecalis*^31^. Quercetin inhibited the growth of *P. mirabilis* at 1024 μg mL^−1^, and *P. aeruginosa* at 500 µg mL^–1^^32,33^. The MIC and MBC of quercetin against SM were found to be at a lower concentration, indicating that the minimum concentration of a drug is adequate to inhibit the growth of the organism. Antibiofilm agents will restrict pathogenic adhesion, and the resulted free-living cells may easily be confronted with any antibiotic use or the host immune system^34^. Thus, in the last decade, the need for new antibiofilm drugs and research focusing on their molecular mechanisms has risen. In this study, dose-dependent anti-biofilm capacity of quercetin was evaluated with the help of crystal violet biofilm assay. Up to 63% biofilm suppression of SM was detected with quercetin treatment. Further, MTT assay was performed to assess the decreased mitochondrial dehydrogenases and metabolically active cells. The MTT reduction test corroborated the results of the MBC assay, indicating the detrimental effects on cell viability with quercetin exposure.

The occurrence of flagella, pili and fimbriae promotes bacterial motility and biofilm formation. The fimbriae of SM augments biofilm development among air–liquid boundaries. SM motility mechanisms promote the early attachment of pathogens to the host and maintain internal colonization during pathogenesis. Hence, we evaluated the outcome of quercetin on the motility of SM*,* and the results revealed that quercetin could strongly distress the flagella assisted swarming motility. In addition, the inhibitory effect of quercetin on QS mediated virulence determinants in SM was greater than the effect reported for non-natural QS inhibitors. Furthermore, selection pressure on microbes has been observed with antibiotics, leading to the development of drug resistance. Thus, quercetin antibiofilm capacity was tested against SM at sub-MIC concentrations, which demonstrated significant concentration-dependent activity. Consistent with this report, Ouyang et al*.* found a decrease in biofilm formation of *P. aeruginosa* in the presence of quercetin compared to that of untreated ones^14^. In addition, our findings are in accordance with those of Packiavathy et al., who reported that biofilm formation by foodborne pathogens treated with methyl eugenol (10 μg mL^−1^) is scarce compared with that of untreated pathogens^35^. Further to understand the anti-QS and anti-biofilm potential of quercetin at molecular level and to support the outcome of in vitro results, the real-time PCR analysis was performed. Quercetin was found to down regulate the expression of both flbC and flbD (flagellar transcriptional regulators). BsmA and BsmB are the important transcription factors for enhancing the production of SM type I pilus and are also inhibited. Bacterial metabolic retorts to antimicrobial agents have not been well inspected with cutting-edge metabolomics, and decoding the metabolome of pathogenic bacterial communities can possibly lead to ground-breaking tactics for active antibacterial therapy. Hence, in the present study, global metabolic changes in SM were investigated following exposure to quercetin. Our results show, for the first time, that quercetin induces common global metabolic alterations in SM. During quercetin treatment, metabolism of organic acids and amino sugars and energy pathways altered significantly. All bacterial metabolites that significantly differed (P < 0.05) in quercetin treatment compared to controls were identified. This endorsed us to recognize the most noticeable variations accompanying a precise metabolic pathway subsequently plotted on metabolism as defined by KEGG database analysis. Metabolites in fatty acid metabolism, amino acid metabolism, the pentose phosphate pathway, purine metabolism and pyrimidine metabolism were found to suggestively differ between control and treated cells. A variety of human and animal pathogens were examined with the help of a pathogen and host interaction model, *C.elegans*. This is the first study, to the bestofour knowledge, to detail the development of a *C.elegans* model to determine the nematode response to quercetin-mediated defense against pathogenic infection. Relating the in vitro assays with the in vivo* C. elegans* rescue assay, it has become clear that quercetin rescues *C. elegans* from uropathogen infection by interfering with the bacterial QS mechanism, which could reduce their associated virulence, such as adhesion. With the reduced adhesive effect of the uropathogen SM, the amount of colonization in the *C. elegans* intestine was reduced, which reflects a reduction in pathogenesis.
